# Protocol for developing a core outcome set for traditional Chinese medicine in the treatment of pharyngitis

**DOI:** 10.3389/fmed.2026.1859133

**Published:** 2026-07-17

**Authors:** Danni Li, Chenyao Zhang, Boya Wei, Yanjun Jin, Xinyao Jin, Fengwen Yang, Wentai Pang, Junhua Zhang

**Affiliations:** 1 Evidence Based Medicine Center, Tianjin University of Traditional Chinese Medicine, Tianjin, China; 2 National Key Laboratory of Modern Chinese Medicine Creation, Tianjin, China

**Keywords:** core outcome set, Delphi technique, integrated traditional Chinese and Western medicine, pharyngitis, protocol

## Abstract

**Background:**

Pharyngitis, a prevalent upper respiratory tract disorder, significantly impairs patients’ health-related quality of life due to persistent symptoms such as sore throat and dryness. In particular, the recurrent and lingering nature of chronic cases places a substantial strain on both the healthcare system and society. Although conventional therapies dominated by anti-infective and anti-inflammatory agents are widely employed, their efficacy in curbing recurrence and enhancing long-term prognosis remains suboptimal. Conversely, Traditional Chinese Medicine (TCM) has demonstrated potential advantages in modulating the local inflammatory microenvironment and regulating systemic function, leading to a rapid proliferation of related randomized controlled trials (RCTs). Nevertheless, existing clinical evidence is plagued by significant heterogeneity in the selection and reporting of outcome measures. A substantial number of studies excessively rely on ill-defined composite endpoints, such as the “total effective rate,” or utilize *ad hoc* symptom scales lacking validation for reliability and validity, thereby precluding high-quality quantitative assessment. Such non-standardized and fragmented evaluation criteria severely impede cross-study evidence synthesis and comparison, consequently restricting the formulation of high-level evidence-based clinical guidelines.

**Objectives:**

The primary objective of this protocol is to develop a Core Outcome Set (COS) specifically tailored for clinical trials of Traditional Chinese Medicine (TCM) in the treatment of pharyngitis. This process will strictly adhere to the international standards established by the Core Outcome Measures in Effectiveness Trials (COMET) Initiative, employing a systematic methodological framework that integrates diverse stakeholder perspectives.

**Methods:**

The development process will unfold in four distinct phases. First, a systematic review will be conducted to comprehensively capture all previously reported outcomes in pharyngitis research, thereby establishing an initial item pool. Second, qualitative research comprising semi-structured interviews will be utilized to uncover outcomes deemed critical by patients and frontline clinicians that may have been overlooked in existing literature. Third, a multidisciplinary panel—including TCM and Western medicine practitioners, pharmacologists, methodologists, and patient representatives—will participate in a two-round Delphi survey to anonymously rate the importance of these outcomes. Finally, a face-to-face consensus meeting employing the modified Nominal Group Technique (NGT) will be convened to vote on and ratify the final COS items, along with their definitions and recommended measurement instruments.

**Discussion:**

The establishment of this COS is expected to standardize outcome reporting in clinical trials regarding TCM for pharyngitis. By mitigating selective reporting bias and facilitating evidence synthesis, this COS will support high-quality guideline development, ultimately enhancing both research integrity and the standard of patient care.

**Clinical trial registration:**

https://www.comet-initiative.org/Studies/Details/2601, identifier 2601.

## 1 Background

A Core Outcome Set (COS) represents a standardized collection of essential outcomes, determined through consensus, that mandates consistent measurement and reporting across all clinical trials addressing a specific health condition (1). The adoption of a COS is pivotal for normalizing outcome reporting, thereby enhancing the overall caliber of trials and facilitating the valid comparison, contrast, and pooling of data—such as in systematic reviews and meta-analyses—which ultimately serves to curtail avoidable research waste (2). In recent years, the successful development of COS for various conditions, including COVID-19 (3, 4) and chronic pain (5), has led to their progressive integration into clinical research practice.

Pharyngitis constitutes a highly prevalent upper respiratory tract infection characterized by inflammation of the pharyngeal mucosa and associated lymphoid structures (6). This condition imposes a substantial global disease burden, not only by severely compromising patients’ health-related quality of life (HRQoL) but also by precipitating significant healthcare resource consumption and productivity losses (7). In the United States, this condition represents approximately 1–2% of annual outpatient clinic visits, underscoring its significant strain on medical resources (8). The etiology of pharyngitis is diverse. Viral infections are the predominant cause of acute pharyngitis, accounting for approximately 50–80% of cases, while Group A beta-hemolytic streptococcus (GABHS) remains the primary bacterial pathogen (9). More precisely, this pathogen is responsible for an estimated 5.2 million yearly outpatient encounters nationwide, demonstrating a distinct seasonal surge in incidence throughout the winter (10). In contrast, chronic pharyngitis is often associated with persistent or recurrent mucosal irritation, which may be related to environmental exposure, long-term vocal overuse, smoking, alcohol consumption, or other non-infectious contributing factors.

For pharyngitis, which dominates the clinical landscape, modern medicine currently lacks specific curative treatments, relying instead on supportive care strategies such as hydration and analgesia (e.g., non-steroidal anti-inflammatory drugs) (9). Conversely, Traditional Chinese Medicine (TCM)—historically referred to as “Houbi” in this context—boasts a millennia-long legacy in managing this condition. Anchored in a unique theoretical framework, numerous herbal formulas and proprietary Chinese medicines (particularly those aimed at clearing heat, detoxifying, and soothing the throat) have demonstrated potential in alleviating throat pain and shortening disease duration, establishing TCM as a vital complementary and alternative therapeutic option in East Asia and globally (11).

However, with the increasing number of clinical studies evaluating TCM interventions for pharyngitis, important methodological limitations in outcome selection and reporting have become apparent. In particular, many studies continue to rely heavily on broadly defined efficacy indicators such as “total effective rate,” for which the criteria used to define clinical effectiveness often vary substantially across studies. Symptom-related outcomes are also assessed using heterogeneous scoring systems, including visual analog scales, categorical symptom grades, self-developed symptom scores, and TCM syndrome scores, with inconsistent definitions, scoring ranges, and assessment time points. Moreover, the reported outcomes are highly diverse, ranging from patient-reported symptoms, such as sore throat and pharyngeal discomfort, to clinician-assessed signs, such as the degree of pharyngeal congestion, and laboratory markers, such as C-reactive protein levels (12). This fragmented approach to outcome reporting limits valid cross-study comparisons and data integration, thereby creating a significant barrier to translating research evidence into reliable clinical practice.

Consequently, there is an imperative need to develop a dedicated COS specifically tailored for clinical trials investigating TCM for pharyngitis. Such an initiative is essential for elevating research quality and enhancing the comparability and synthesis efficiency of evidence in this field. Herein, we delineate the methodological framework proposed to define this COS and outline the subsequent plans for its dissemination and implementation.

This study is a protocol and therefore aims to describe the planned methodology for developing a core outcome set, rather than to report the final results of the completed COS development process.

## 2 Methods

**FIGURE 1:**
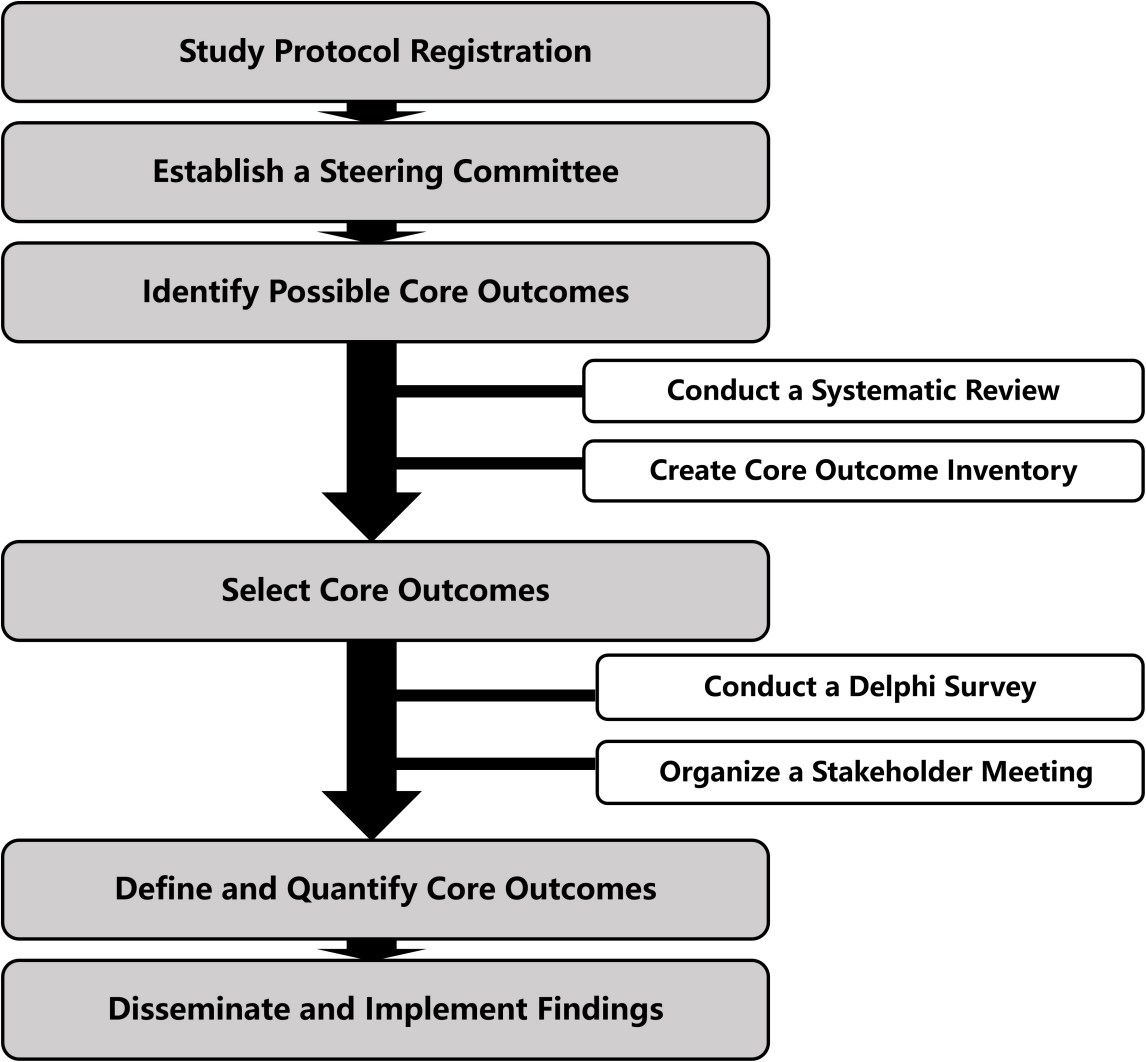
The stage of developing the core indicator set for clinical trials of traditional Chinese medicine treatment of pharyngitis.

The developmental framework of this COS is fundamentally grounded in the methodological standards delineated within the handbook of the Core Outcome Measures in Effectiveness Trials (COMET) Initiative (1). Recognized for its systematic rigor and robustness, this methodological approach has been successfully validated through its application in constructing COS for various conditions within the context of Traditional Chinese Medicine (TCM), including knee osteoarthritis (13) and functional constipation (14). To guarantee transparency and structural integrity, the design and reporting of this protocol will meticulously align with the guidelines set forth in the Core Outcome Set-STAndardized Protocol Items (COS-STAP) Statement (15). A schematic representation of the multi-stage development process is illustrated in Figure 1.

As of this manuscript’s submission, the study remains strictly in the protocol phase, meaning no empirical data have yet been generated. The protocol has been formally registered, with the specific registration identifier detailed in the subsequent section. Our projected research trajectory encompasses several sequential phases: convening a steering committee in May 2026; executing a systematic review to map potential core outcomes between June and August 2026; and formulating a preliminary core outcome inventory from September to October 2026. The consensus process will then proceed via a Delphi survey (November 2026 to January 2027), followed by a stakeholder meeting in February 2027. By March 2027, the core outcome set (COS) will be finally defined and quantified. Consequently, none of these post-registration activities have commenced. Participant recruitment for the consensus panels is slated for completion by October 2026, while overall data acquisition will conclude by March 2027. Written informed consent will be obtained from all participants before participation. Broad dissemination and implementation of the finalized findings will commence from April 2027 onward.

## 3 Registration

The protocol for this initiative has been officially indexed within the Core Outcome Measures in Effectiveness Trials (COMET) database (Registration Number:2601).

## 4 Expert steering group (ESG)

Oversight for the development of this COS will be provided by a multidisciplinary Expert Steering Group (ESG), established to guide the strategic direction of the project.

We aim to curate a diverse panel of authorities to constitute the ESG. Its membership will span critical disciplines, including Traditional Chinese Medicine (TCM) laryngology, conventional otorhinolaryngology, clinical epidemiology, evidence-based medicine methodology, and Chinese herbal pharmacology, alongside patient representatives. This comprehensive composition ensures that senior specialists from every relevant sector contribute a holistic professional perspective.

The primary mandate of the ESG entails:

- Conducting a rigorous methodological audit and refinement of the overarching study protocol.
- Collaborating to define the inclusion criteria and finalizing the roster for the Stakeholder Group.
- Auditing, deliberating upon, and ratifying the preliminary inventory of outcome measures to be utilized in the Delphi consensus process.

Furthermore, ESG members will spearhead the pilot phase of the first-round Delphi survey to optimize the questionnaire design. Subsequently, these experts will be integrated into the Stakeholder Group as core contributors to complete the full iteration of the survey process.

Notably, patient representatives will play a pivotal role in scrutinizing the terminology to ensure plain language and readability, as well as facilitating engagement and dissemination within the patient community. To guarantee operational efficiency, a dedicated management unit will be established within the ESG to oversee daily logistics and ensure steady project progression.

## 5 Scope of the core outcome set (COS)

Pharyngitis is defined clinically as an inflammatory condition affecting the pharyngeal mucosa, submucosal tissues, and associated lymphoid structures. Within the paradigm of Traditional Chinese Medicine (TCM), this condition is typically categorized into distinct syndromes based on symptomatic presentation (e.g., pharyngeal pain, dryness, itching, or the sensation of a foreign body).

This COS is designed to be applicable to clinical trials evaluating TCM interventions for pharyngitis—encompassing both acute and chronic forms—across all demographic cohorts, including pediatric and adult populations. The scope remains inclusive, imposing no preliminary restrictions regarding specific TCM syndromes, disease duration, or severity levels.

In this context, “TCM interventions” specifically refer to internal therapies guided by TCM theory. This primarily encompasses herbal compound prescriptions (such as decoctions, granules, and pills) and proprietary Chinese medicines (including tablets, capsules, and oral liquids). External therapies, such as acupuncture and Tuina (massage), are excluded from the current framework.

Furthermore, this COS is intended to capture outcomes assessed during the active treatment phase, at the end of treatment, and throughout the post-treatment follow-up period. Considering the differences in disease course and clinical objectives between acute and chronic pharyngitis, follow-up time points and follow-up-related outcomes will be considered and interpreted separately for the two conditions. For acute pharyngitis, outcome assessment will primarily focus on symptom resolution, clinical recovery, and complication-related outcomes during or shortly after treatment. For chronic pharyngitis, greater emphasis will be placed on sustained symptom improvement, recurrence or relapse, acute exacerbation, quality of life, patient-reported outcomes, and other clinically relevant longer-term outcomes.

It is crucial to clarify that the objective of this initiative is not to standardize diagnostic criteria for pharyngitis or to regulate TCM treatment protocols; rather, the exclusive focus is on establishing consensus regarding “what outcomes to measure.”

## 6 Identification of candidate outcome measures

To compile an exhaustive inventory of candidate outcomes, we will execute a systematic literature review. This process aims to aggregate all outcome measures previously reported in clinical inquiries regarding TCM interventions for pharyngitis.

### 6.1 Eligibility criteria

To guarantee the homogeneity and methodological integrity of the studies selected, we have established rigorous screening protocols.

#### 6.1.1 Inclusion criteria

- Study Design: Randomized controlled trials (RCTs), quasi-randomized controlled trials, and prospective observational studies will be included.
- Population: Patients clinically diagnosed with acute pharyngitis or common chronic pharyngitis will be eligible, without restrictions on age, sex, ethnicity, disease severity, or clinical setting. In this protocol, common chronic pharyngitis refers to chronic pharyngitis diagnosed as the primary condition, rather than pharyngeal symptoms secondary to specific etiologies or independent disease entities. Outcomes for acute and chronic pharyngitis will be extracted and classified separately where possible. Studies involving mixed populations will be included only when pharyngitis-related outcomes can be clearly identified and separately extracted.
- Intervention: The experimental group must utilize internal TCM therapies as the primary treatment modality. This encompasses, but is not limited to, herbal compounds, single-herb extracts, or oral proprietary Chinese medicines.
- Outcome: Studies reporting at least one clinical outcome related to pharyngitis, such as symptoms, physical signs, inflammatory or infection-related indicators, recurrence, quality of life, TCM syndrome-related outcomes, patient-reported outcomes, or safety outcomes, will be included.

#### 6.1.2 Exclusion criteria

- Non-primary Evidence: Systematic reviews, meta-analyses, editorial commentaries, expert opinions, and non-empirical correspondence are excluded.
- Design Limitations: Retrospective studies and case series with insufficient sample sizes (*n* < 10) will be omitted.
- Non-clinical Research: Animal models and *in vitro* cellular experiments are strictly ineligible.
- Publication Type: Conference proceedings containing only abstracts without accessible full-text data will not be included.
- Studies will be excluded if they focus primarily on pharyngeal symptoms caused by specific etiologies or independent disease entities rather than acute pharyngitis or common chronic pharyngitis as the primary condition. These include, but are not limited to, gastroesophageal reflux disease/laryngopharyngeal reflux, obstructive sleep apnea-hypopnea syndrome, occupational exposure-related pharyngeal symptoms, radiotherapy- or chemotherapy-induced pharyngeal symptoms, allergy, postnasal drip, chronic tonsillitis, laryngitis, tumors, or other clearly defined systemic or local diseases.

Note: To minimize publication bias, no linguistic restrictions will be applied during the screening phase.

### 6.2 Search strategy and data sources

The literature retrieval protocols adhere to the methodological specifications aimed at developing Core Outcome Sets (COS) as outlined by the COMET initiative (1). Our objective is to construct a bilingual retrieval repository that comprehensively spans both English and Chinese literature.

#### 6.2.1 Database selection

We will conduct a systematic query across major biomedical databases in both languages:

- English Repositories: PubMed, Embase, The Cochrane Library, and Web of Science.
- Chinese Repositories: Given the specific context of Traditional Chinese Medicine, we will prioritize searches within the China National Knowledge Infrastructure (CNKI), Wanfang Data, the VIP Database for Chinese Technical Periodicals, and the Chinese Biomedical Literature Service System (SinoMed) (16).

#### 6.2.2 Search term construction

The retrieval logic employs a hybrid approach combining “Medical Subject Headings (MeSH)” with free-text keywords, synthesized using Boolean operators.

- Disease-related terms: Including but not limited to “Pharyngitis,” “Sore throat,” and their Chinese equivalents such as “Yan Yan” and “Hou Bi.”
- Intervention-related terms: Encompassing terminology such as “Traditional Chinese Medicine,” “Herbal medicine,” “Phytotherapy,” and related terms like “internal therapy” or “proprietary Chinese medicine.”

### 6.3 Literature screening and selection process

The screening workflow will be governed by the PRISMA flowchart, adhering to the following specific procedures:

(1) Deduplication: All retrieved citations will be imported into bibliographic management software (e.g., EndNote), where duplicate entries will be initially purged.

(2) Preliminary Screening: Two investigators will independently review titles and abstracts to discard clearly irrelevant studies based on the pre-defined eligibility criteria.

(3) Full-text Assessment: Manuscripts clearing the initial screen will be retrieved in full. The same two investigators will independently conduct a detailed review to confirm final eligibility.

(4) Conflict Resolution: Should discrepancies arise at any stage of the screening, resolution will first be attempted through consultation between the two reviewers. If a consensus cannot be reached, a third senior researcher will be consulted to arbitrate and make the final decision.

### 6.4 Data acquisition and outcome inventory compilation

We intend to design and validate a standardized electronic template (via Microsoft Excel) to facilitate data capture. Two investigators will independently undertake the abstraction of the following variables: bibliographic details (authorship, publication year), methodological design, cohort size, subject demographics (e.g., age profiles, pharyngitis classification), specifics of the TCM and comparator interventions, alongside every reported outcome measure.

Crucially, we will capture the precise nomenclature of the outcomes, their definitions (where available in the primary text), and the specific timeframes of assessment.

All distinct outcomes identified through this process will be aggregated into a preliminary “outcome repository.” To streamline the review by the steering committee and facilitate the subsequent Delphi exercise, We will map these items according to the seven-category classification framework for outcomes in traditional Chinese medicine clinical research proposed by Zhang Junhua et al. (17), while integrating the clinical nuances of pharyngitis and the specific attributes of TCM interventions. Consequently, outcomes will be stratified into the following functional domains:

- Symptoms and local signs: Encompassing the assessment of pharyngeal pain, pyrexia, mucosal hyperemia, siccity or burning sensations in the throat, tussis, and other associated physical manifestations.
- Exploratory laboratory and biomarker outcomes: Incorporating systemic inflammatory markers such as tumor necrosis factor-alpha (TNF-α), interleukin-6 (IL-6), and C-reactive protein (CRP), alongside white blood cell (WBC) counts and additional diagnostic metrics.
- Traditional Chinese Medicine-related outcomes: Focusing on the clinical response rates of Traditional Chinese Medicine (TCM) patterns, quantitative syndrome scoring systems, and relevant holistic efficacy criteria.
- Life impact and patient-reported outcomes: Assessing multidimensional domains such as emotional well-being, Brief Pain Inventory (BPI) metrics, occupational functionality, social engagement, and overall life satisfaction.
- Resource use and economic outcomes: Evaluating the temporal aspects of recovery (time to cure), cost-effectiveness ratios (CER), indirect economic burdens, direct pharmaceutical expenditures (C), and related financial impact indicators.
- Safety outcomes: Documenting the incidence and severity of adverse drug reactions (ADRs) and unexpected clinical events.
- Recurrence and prognosis-related outcomes: Primarily focusing on the evaluation of recurrence rates, the frequency of relapse, and the long-term maintenance of therapeutic efficacy.

### 6.5 Review and refinement

The consolidated outcome repository will undergo a stringent vetting process by the Expert Steering Group (ESG). The primary objective of this audit is to curtail redundancy through the amalgamation of synonyms—for instance, merging terms like “pharyngeal pain” and “throat soreness”—and the synthesis of highly correlated concepts. This filtration process ensures that the definitive roster submitted to the stakeholder panel remains lucid, succinct, and devoid of duplication.

Following formal endorsement by the Expert Steering Group (ESG), the consolidated data will be migrated to the dedicated core outcome set repository—a collaborative platform established by Tianjin University of Traditional Chinese Medicine and ChiCOS—where the built-in digital modules will facilitate the Delphi survey distribution (18). Crucially, the precise nomenclature for all outcomes will be finalized in consensus with our patient representatives to guarantee a patient-centric orientation and optimal comprehensibility.

The preliminary candidate outcome lists for acute pharyngitis and chronic pharyngitis have been provided as Supplementary Tables 1, 2, respectively, and will serve as the basis for the first-round Delphi questionnaire.

## 7 Derivation of the core outcome set

We intend to deploy a modified Delphi strategy to crystallize the final set of core outcomes. This methodology constitutes a well-established consensus-building mechanism involving iterative rounds of anonymous surveying to synthesize expert opinion (1). Uniquely, this approach empowers participants to articulate independent viewpoints—insulated from peer pressure—while gradually converging toward consensus through controlled feedback loops.

### 7.1 Recruitment of the stakeholder panel

Initially, the ESG will propose a slate of potential candidates for the stakeholder panel. Furthermore, to ensure diversity, we plan to orchestrate a broad dissemination campaign to identify and enlist a wider array of participants. Outreach avenues will encompass internal newsletters and digital platforms of relevant professional bodies, such as:

- The China Association of Chinese Medicine (CACM) and its specialized Otorhinolaryngology Committee.
- The World Federation of Chinese Medicine Societies (WFCMS).
- Other pertinent academic platforms integrating Traditional Chinese and Western otolaryngology.
- Prominent health communities and forums dedicated to patient advocacy.

### 7.2 Sample size and composition

Currently, there exists no universally standardized statistical axiom for computing the requisite sample size for Delphi studies. However, retrospective analyses of health science literature indicate that panel sizes typically oscillate between 15 and over 100 participants (19).

Our objective is to secure a minimum of 15 contributors for each core stakeholder domain. We will guarantee that the panel incorporates representation from at least three pivotal categories:

(1) Patient Representatives: Individuals with a lived experience of acute or chronic pharyngitis, or their caregivers.

(2) Clinical Practitioners: Physicians possessing extensive proficiency in the management of pharyngitis (encompassing, but not limited to, TCM laryngologists, conventional ENT surgeons, and general practitioners).

(3) Researchers and Methodologists: Spanning clinical trialists, experts in Chinese herbal pharmacology, and specialists in evidence-based methodology.

### 7.3 Enrollment procedure

Prospective contributors may signal their intent to participate via a designated online portal. The project management unit will scrutinize applications based on professional credentials, clinical expertise, or patient experience to finalize the panel composition, thereby ensuring equilibrium across groups.

Should a deficit in participant numbers arise within any specific stakeholder category, the steering group will reactivate and expand the recruitment perimeter to guarantee broad representativeness in the consensus process.

## 8 The Delphi survey procedure

Following the preparatory phase, we will disseminate plain-language instructional materials and access links for the electronic questionnaire to all identified stakeholders via email, formally inviting them to engage in the Delphi process. As previously detailed, the participant roster encompasses clinical specialists in both TCM and conventional otorhinolaryngology, methodologists, TCM researchers, and patient representatives. We have selected the Delphi technique as it is a proven, structured instrument for deriving consensus among professionally diverse and geographically scattered groups (20).

The inquiry will capture the demographic profiles of the respondents, alongside their specific professional expertise or lived experience regarding the TCM management of pharyngitis.

We will enforce a “quasi-anonymous” protocol to govern the process:

1. Anonymization: Each participant will be assigned a unique alphanumeric identifier, devoid of any personally identifiable information (PII) such as names or telephone numbers.

2. Access Control: Access to the master key linking contact details to these identifiers will be strictly restricted to designated members of the central research management unit.

3. Operational Use: The exclusive utility of this master list is to facilitate the transmission of personalized prompts to non-responders or to conduct follow-up regarding their feedback if necessitated.

The Delphi exercise is projected to span a minimum of two iterations.

Round 1

In the inaugural round, participants will be requested to rate the criticality of the preliminary outcome inventory (generated via the antecedent literature review and interviews). This procedure adheres to the standardized workflow advocated by the COMET (Core Outcome Measures in Effectiveness Trials) initiative (1).

We will employ the internationally recognized 9-point Likert scale, originally formulated by the GRADE (Grading of Recommendations Assessment, Development and Evaluation) working group. This metric is specifically calibrated to assess the importance of outcomes for decision-making and is widely adopted in COS scholarship (21).

- Scoring Matrix: 1–3 signifies “limited importance”; 4–6 denotes “important but not critical”; and 7–9 represents “critical significance.”
- New Nominations: Concurrently, respondents will be afforded the opportunity to nominate novel outcome measures via open-ended text fields if they perceive any significant omissions.
- Post-Round Processing: Upon conclusion of Round 1, the expert panel will vet, amalgamate synonyms for, and standardize any newly proposed items before incorporating them into the inventory for the subsequent round.

Round 2

In the second iteration, participants will be presented with the comprehensive list, comprising both the initial outcomes and those generated in Round 1. To augment their judgment, each participant will be provided with specific feedback metadata:

1. The rating they individually assigned in the previous round;

2. The aggregate distribution of scores within their specific stakeholder peer group (e.g., “Clinical Expert Group” vs. “Patient Group”).

Participants will then be invited to re-appraise and score the importance of all outcome measures. We will apply pre-defined thresholds, derived from rigorous consensus definitions recommended in systematic reviews (22), to determine inclusion or exclusion:

- Consensus for Inclusion (Core Outcome): Greater than 70% of participants in every stakeholder stratum rating the item as “critical” (7–9), AND less than 15% in any stratum rating it as “limited importance” (1–3).
- Consensus for Exclusion: Greater than 70% of participants in every stakeholder stratum rating the item as “limited importance” (1–3), AND less than 15% in any stratum rating it as “critical” (7–9).
- Equivocal (No Consensus): Any scenario failing to satisfy the aforementioned criteria for either inclusion or exclusion.

Following the closure of Round 2, the International Steering Group (ISG) will review the Delphi results, with particular attention to items classified as “equivocal” or “no consensus,” as well as any differences in ratings across stakeholder groups. Items not meeting the predefined criteria for either inclusion or exclusion will be classified as “equivocal” or “no consensus” and will be further reviewed by the ISG. A third Delphi round will be initiated if clinically or methodologically important outcomes remain without consensus after Round 2, if substantial disagreement persists between stakeholder groups, or if newly suggested outcomes require further rating before the final consensus meeting. If required, the third round will focus primarily on unresolved items rather than all outcomes previously assessed. Items that have already met the predefined criteria for inclusion or exclusion will not be re-rated unless the ISG identifies a compelling methodological reason.

Each survey window will remain open for 4 weeks. To minimize non-response, personalized email reminders will be sent to participants who have not completed the questionnaire, with reminders scheduled at approximately one and 3 weeks after the initial survey invitation. If the response rate is below the predefined acceptable threshold, the survey window may be extended by one to 2 weeks, and additional reminders may be issued. A response rate of at least 70% in each Delphi round will be considered acceptable. To maintain consistency in feedback and scoring between rounds, participants who complete a given round will be invited to participate in the subsequent round. Participants who do not respond to a given round will be excluded from the denominator for that round’s analysis. Attrition between rounds, including the number and proportion of non-responders in each stakeholder group, will be recorded and reported.

Statistical analyses will be performed using SPSS software, version 26.0. Results will be summarized using frequencies, percentages, medians, and interquartile ranges (IQRs), as appropriate.

## 9 Consensus meeting

Stakeholders who have successfully navigated the complete Delphi trajectory will be invited to convene for a definitive Consensus Meeting. To optimize attendance logistics and maximize participation, this session will be orchestrated via a hybrid modality, integrating physical presence with virtual video conferencing.

The pivotal objective of this assembly is to adjudicate the final status of outcome measures that remained classified as “equivocal” (no consensus) following the Delphi iterations.

To facilitate this structured dialogue, we will leverage the Modified Nominal Group Technique (NGT). The NGT is a validated methodology designed to ensure an egalitarian platform where all voices—spanning patient representatives to senior experts—can articulate viewpoints without hierarchy. It is instrumental in identifying divergence and fostering consensus through a non-adversarial process (23), having been successfully deployed in the development of numerous COS protocols.

During the session, any outcome measure that fails to secure the criteria for “consensus for inclusion” following exhaustive deliberation will be definitively relegated to “consensus for exclusion” status and omitted from the core set. The ultimate deliverable of this meeting will be the ratified “COS for TCM treatment of pharyngitis.”

## 10 Measurement of core outcomes (phase 2)

Having crystallized the “what to measure” component (the final COS list), the initiative will transition to Phase 2: establishing the “how to measure” parameters.

The mandate of this phase is to formulate precise operational definitions for each core outcome and to endorse specific Outcome Measurement Instruments (OMIs).

We will initiate a systematic reconnaissance to harvest potential definitions and measurement tools from the following repositories:

1. Clinical Practice Guidelines: Both national and trans-national guidelines governing the management of pharyngitis (covering TCM and Western medicine).

2. Evidence Synthesis: Published Cochrane systematic reviews and clinical trials, with a specific emphasis on TCM inquiries.

3. Authoritative Databases: Recognized nomenclature initiatives and outcome registries.

All retrieved candidate OMIs will undergo a structured appraisal by the project team. In accordance with the COMET initiative and the COSMIN (COnsensus-based Standards for the selection of health Measurement INstruments) framework, we will evaluate the methodological quality and measurement properties of each candidate instrument, with particular emphasis on content validity. Other measurement properties to be considered will include construct validity, criterion validity where applicable, internal consistency, reliability, measurement error, responsiveness, interpretability, and feasibility (24). The appraisal will also consider whether each instrument is appropriate for the target population, stakeholder perspective, clinical setting, disease characteristics of acute or chronic pharyngitis, and TCM-related concepts, such as TCM syndrome assessment where relevant.

Subsequently, a secondary consensus assembly—comprising methodologists, dual-trained clinicians (TCM and Western medicine), and patient representatives—will be convened. This group will review the evidentiary assessment to ratify the optimal definition and preferred instrumentation for each core outcome.

## 11 Dissemination and implementation strategy

The deployment and broad propagation of the finalized COS inventory—encompassing specific outcome items, operational definitions, and endorsed measurement instruments—will be championed by the International Steering Group (ISG).

Our dissemination architecture is structured around the following pillars:

1. Scholarly Publication: We intend to codify the complete developmental trajectory and the definitive findings into a comprehensive manuscript. This will be submitted for publication in prestigious peer-reviewed periodicals specializing in either Traditional Chinese Medicine or conventional otolaryngology.

2. Conference Engagement: Results will be showcased via podium presentations and academic poster sessions at relevant domestic and international symposia (e.g., gatherings hosted by the China Association of Chinese Medicine or the World Federation of Chinese Medicine Societies).

Furthermore, we will engage in active liaison with the Otorhinolaryngology Head and Neck Surgery Branch of the Chinese Medical Association, pertinent regulatory authorities (such as the Center for Drug Evaluation [CDE]), and major patient advocacy coalitions.

The ultimate objective is to advocate for the formal adoption of this COS as the “minimum reporting standard” for future clinical trials and systematic reviews in this domain. This strategic alignment aims to significantly augment research caliber and ensure cross-study comparability (25).

## 12 Discussion

The construction of this Core Outcome Set (COS) for TCM treatment of pharyngitis serves to furnish a definitive, authoritative benchmark for future clinical inquiries. We anticipate that the systematic deployment of this COS across clinical trials, prospective cohorts, and systematic reviews will fundamentally catalyze an improvement in the standardization, relevance, and comparability of research within this domain, ultimately translating into optimized clinical practice and tangible patient benefits (26).

### 12.1 Standardizing research criteria: elevating quality and comparability

Currently, clinical trials investigating TCM for pharyngitis suffer from marked discordance in outcome selection. Specifically, the lack of consensus on evaluating nuanced TCM-specific constructs significantly impedes cross-study data comparison and evidence synthesis. The establishment of this COS directly resonates with the mandates of the SPIRIT (Standard Protocol Items: Recommendations for Interventional Trials) statement (1).

By harmonizing the dynamic assessment of pivotal symptoms, physical signs, and TCM syndromes, this COS will drastically enhance the comparability between studies evaluating diverse interventions (e.g., various herbal formulae or proprietary medicines). This standardization will underpin a robust foundation for high-caliber systematic reviews—particularly Network Meta-Analyses leveraging Individual Patient Data (IPD) (27)—thereby enabling a more precise appraisal of the relative efficacy of different TCM modalities.

### 12.2 Mitigating reporting bias: fortifying the veracity of evidence synthesis

Outcome reporting bias constitutes a critical bottleneck that has long compromised the reliability of TCM evidence. Echoing the momentum driven by the CROWN (CoRe Outcomes in Women’s and Newborn health) initiative (28), the adoption of COS has become a hallmark of quality in international high-impact journals.

This COS aims to compel academic periodicals—especially those bridging TCM and Western medicine—to mandate the disclosure of the full spectrum of core outcomes, rather than permitting the selective presentation of non-core or surrogate endpoints solely because they yield positive results. Such a transparent reporting mechanism will effectively curtail selective reporting bias, significantly bolstering the overall veracity and credibility of the TCM clinical evidence base (1).

### 12.3 Supporting evidence-based decision making: informing guideline development

Evidence-based clinical guidelines act as the pivotal conduit for integrating TCM services into modern healthcare architectures. Guideline development bodies, including the World Health Organization (WHO), rely heavily on high-quality evidence synthesis when formulating recommendations (29).

Through the application of this COS, efficacy data specific to distinct TCM patterns (e.g., Wind-Heat pattern or Lung-Dryness pattern) will become more lucid and consistent. This clarity will empower guideline panels (analogous to institutions like NICE) to formulate more robust clinical advisories based on reliable evidence. Consequently, this will enable practitioners to navigate TCM modalities with greater assurance and standardization in clinical scenarios (1).

### 12.4 Establishing a collaborative ecosystem: infrastructure for international TCM research

The very trajectory of developing this COS—involving iterative Delphi rounds and consensus assemblies—has crystallized an invaluable international nexus of stakeholders. This network aggregates the collective intelligence of TCM experts, otorhinolaryngologists, epidemiologists, methodologists, and patient representatives.

Given the idiosyncrasies of personalized and complex TCM interventions, such a multidisciplinary collaborative infrastructure is indispensable (30). This platform is poised to serve as a springboard for broader scholarly endeavors in the future, such as identifying priority research agendas for pharyngitis, developing TCM-specific Patient-Reported Outcome Measures (PROMs), or driving the international standardization of integrated Chinese-Western diagnostic and therapeutic protocols.

## 13 Limitations

Several limitations of this protocol should be acknowledged. Although the COS will be developed through a structured consensus process involving multiple stakeholders, the final COS will still require further external validation and practical testing in future clinical studies before it can be widely implemented. The applicability, feasibility, and acceptability of the COS across different clinical settings should therefore be further evaluated after its development.

Another limitation is that this protocol focuses on oral Traditional Chinese Medicine therapies for pharyngitis. As a result, the applicability of the developed COS to other TCM interventions, such as acupuncture, external therapies, or combined treatment approaches, may be limited. Future studies may need to further assess whether the COS is suitable for broader TCM intervention modalities.

In addition, acute pharyngitis and chronic pharyngitis have different disease courses and clinical characteristics and have therefore been considered separately in this protocol. Nevertheless, follow-up-related outcomes, especially long-term recurrence, prognosis, and sustained symptom control, are relatively insufficiently reported in the existing literature and may not be fully captured by literature review alone. To address this limitation, stakeholders will be allowed to suggest additional outcomes during the first round of the Delphi survey, and follow-up-related outcomes will be further evaluated and refined during the subsequent consensus process.

## 14 Conclusion

Core Outcome Sets (COS) constitute a fundamental collection of consensus-derived indicators mandated for uniform assessment and reporting across clinical inquiries into specific medical conditions. Under the stewardship of a multidisciplinary steering committee, this initiative focuses on establishing a COS tailored specifically for Traditional Chinese Medicine interventions for pharyngitis. It is anticipated that this project will harmonize future research protocols, bolster the reliability of evidence synthesis, and ultimately confer positive downstream effects on clinical decision-making.
