# Supplementary material for: Protocol for developing a core outcome set for traditional Chinese medicine in the treatment of pharyngitis
Source: Front Med (Lausanne). 2026 Jul 17;13:1859133. doi: 10.3389/fmed.2026.1859133 (PMC13423697; doi:10.3389/fmed.2026.1859133)
Supplement: Supplementary file 1 [file Supplementary_file_1.docx]

# Preliminary Candidate Outcome List for Traditional Chinese Medicine (TCM) Treatment of Acute Pharyngitis

| **Outcome domain** | **Candidate outcomes** |
| --- | --- |
| Symptoms and local signs(21) | Severity of sore throat |
|  | Severity of odynophagia, defined as painful swallowing |
|  | Severity of dysphagia, defined as difficulty swallowing |
|  | Severity of pharyngeal dryness |
|  | Severity of pharyngeal itching |
|  | Severity of pharyngeal burning sensation |
|  | Severity of pharyngeal foreign-body sensation |
|  | Severity of dry cough |
|  | Fever or change in body temperature |
|  | Headache severity |
|  | Fatigue or malaise |
|  | Irritability or restlessness |
|  | Appetite loss |
|  | Halitosis |
|  | Degree of pharyngeal mucosal hyperemia |
|  | Degree of pharyngeal mucosal edema |
|  | Degree of uvular edema |
|  | Tonsillar enlargement |
|  | Tonsillar exudate or purulent spots |
|  | Secretion or exudate on the posterior pharyngeal wall |
|  | Tenderness of anterior cervical or submandibular lymph nodes |
| Recurrence, prognosis, and complications(5) | Recurrence rate |
|  | Time to first recurrence |
|  | Re-consultation rate or revisit rate |
|  | Complication rate, including peritonsillar abscess, otitis media, sinusitis, or lower respiratory tract infection |
|  | Hospitalization rate |
| Safety outcomes(6) | Incidence of adverse events (AEs) |
|  | Incidence of serious adverse events (SAEs) |
|  | Treatment discontinuation due to adverse events (AEs) |
|  | Incidence of gastrointestinal adverse events |
|  | Incidence of allergic reactions |
|  | Abnormal liver function or renal function during treatment |
| Life impact and patient-reported outcomes(10) | Health-related quality of life (HRQoL) |
|  | Sleep disturbance due to throat symptoms |
|  | Interference with eating and drinking |
|  | Interference with speaking |
|  | Interference with work, study, or daily activities |
|  | Pain interference with daily life |
|  | Patient global assessment of improvement |
|  | Treatment satisfaction |
|  | Anxiety symptoms |
|  | Depressive symptoms |
| Resource use and economic outcomes(7) | Total treatment cost |
|  | Medication cost |
|  | Direct medical cost |
|  | Indirect cost |
|  | Number of additional health-care visits |
|  | Cost-effectiveness ratio (CER) |
|  | Incremental cost-effectiveness ratio (ICER) |
| Traditional Chinese Medicine-related outcomes(6) | Traditional Chinese Medicine (TCM) syndrome score |
|  | Traditional Chinese Medicine (TCM) syndrome response |
|  | Traditional Chinese Medicine (TCM) single-symptom score |
|  | Traditional Chinese Medicine (TCM) single-symptom response |
|  | Change in tongue appearance |
|  | Changes in pulse condition |
| Microbiological outcomes(3) | Negative conversion or clearance rate of group A Streptococcus (GAS) |
|  | Negative conversion or clearance rate of Epstein-Barr virus (EBV) |
|  | Negative conversion or clearance rate of adenovirus |
| Exploratory laboratory and biomarker outcomes(7) | Complete blood count (CBC), including white blood cell (WBC) count, neutrophil count or percentage, and lymphocyte count or percentage |
|  | C-reactive protein (CRP) level |
|  | High-sensitivity C-reactive protein (hs-CRP) level |
|  | T-lymphocyte subsets, including cluster of differentiation 3 positive T lymphocytes (CD3+ T lymphocytes), cluster of differentiation 4 positive T lymphocytes (CD4+ T lymphocytes), cluster of differentiation 8 positive T lymphocytes (CD8+ T lymphocytes), and cluster of differentiation 4 positive/cluster of differentiation 8 positive T-cell ratio (CD4+/CD8+ ratio) |
|  | Immunoglobulin and mucosal immune markers, including secretory immunoglobulin A (SIgA), immunoglobulin A (IgA), immunoglobulin G (IgG), and immunoglobulin M (IgM) |
|  | T helper 1/T helper 2 cell ratio (Th1/Th2 ratio) |
|  | Inflammatory cytokine levels, including interleukin-1 beta (IL-1β), interleukin-2 (IL-2), interleukin-6 (IL-6), interleukin-8 (IL-8), tumor necrosis factor-alpha (TNF-α), and interferon-gamma (IFN-γ) |

The laboratory and biomarker outcomes are considered exploratory outcomes and may be assessed only when relevant to the study design, intervention mechanism, and available resources. Measurement instruments for these outcomes will be selected in a subsequent stage after consensus is reached on what should be measured.
