# Supplementary material for: Protocol for developing a core outcome set for traditional Chinese medicine in the treatment of pharyngitis
Source: Front Med (Lausanne). 2026 Jul 17;13:1859133. doi: 10.3389/fmed.2026.1859133 (PMC13423697; doi:10.3389/fmed.2026.1859133)
Supplement: Supplementary file 2 [file Supplementary_file_2.docx]

# Preliminary Candidate Outcome List for Traditional Chinese Medicine (TCM) Treatment of Chronic Pharyngitis

| **Outcome domain** | **Candidate outcomes** |
| --- | --- |
| Symptoms and local signs(19) | Severity of pharyngeal foreign-body sensation |
|  | Severity of pharyngeal dryness |
|  | Severity of pharyngeal itching |
|  | Severity of sore throat |
|  | Severity of dry cough |
|  | Severity of pharyngeal burning sensation |
|  | Severity of sticky sputum or phlegm sensation in the throat |
|  | Difficulty expectorating sputum |
|  | Frequency of throat clearing |
|  | Severity of hoarseness |
|  | Severity of morning retching or dry heaving |
|  | Severity of pharyngeal constriction or tightness |
|  | Degree of pharyngeal mucosal dryness |
|  | Degree of pharyngeal mucosal hyperemia |
|  | Degree of pharyngeal mucosal edema |
|  | Degree of pharyngeal mucosal hypertrophy or thickening |
|  | Degree of posterior pharyngeal wall lymphoid follicle hyperplasia |
|  | Degree of pharyngeal secretion adherence or increase |
|  | Degree of lateral pharyngeal band hypertrophy |
| Recurrence and long-term prognosis(5) | Recurrence rate or relapse rate |
|  | Time to first recurrence or relapse |
|  | Frequency of acute exacerbations |
|  | Re-consultation rate or revisit rate |
|  | Long-term symptom control during follow-up |
| Safety outcomes(6) | Incidence of adverse events (AEs) |
|  | Incidence of serious adverse events (SAEs) |
|  | Treatment discontinuation due to adverse events (AEs) |
|  | Incidence of gastrointestinal adverse events |
|  | Incidence of allergic reactions |
|  | Abnormal liver function or renal function during treatment |
| Life impact and patient-reported outcomes(10) | Health-related quality of life (HRQoL) |
|  | Sleep disturbance due to throat symptoms |
|  | Voice-related impact or interference with speaking |
|  | Interference with eating and drinking |
|  | Interference with work, study, or daily activities |
|  | Impact on social functioning |
|  | Patient global assessment of improvement |
|  | Treatment satisfaction |
|  | Anxiety symptoms |
|  | Depressive symptoms |
| Resource use and economic outcomes(8) | Total treatment cost |
|  | Medication cost |
|  | Direct medical cost |
|  | Indirect cost |
|  | Number of follow-up visits or re-consultations |
|  | Long-term treatment cost |
|  | Cost-effectiveness ratio (CER) |
|  | Incremental cost-effectiveness ratio (ICER) |
| Traditional Chinese Medicine-related outcomes(6) | Traditional Chinese Medicine (TCM) syndrome score |
|  | Traditional Chinese Medicine (TCM) syndrome response |
|  | Traditional Chinese Medicine (TCM) single-symptom score |
|  | Traditional Chinese Medicine (TCM) single-symptom response |
|  | Change in tongue appearance |
|  | Changes in pulse condition |
| Exploratory laboratory and biomarker outcomes(7) | Complete blood count (CBC), including white blood cell (WBC) count, neutrophil count or percentage, and lymphocyte count or percentage |
|  | C-reactive protein (CRP) level |
|  | High-sensitivity C-reactive protein (hs-CRP) level |
|  | T-lymphocyte subsets, including cluster of differentiation 3 positive T lymphocytes (CD3+ T lymphocytes), cluster of differentiation 4 positive T lymphocytes (CD4+ T lymphocytes), cluster of differentiation 8 positive T lymphocytes (CD8+ T lymphocytes), and cluster of differentiation 4 positive/cluster of differentiation 8 positive T-cell ratio (CD4+/CD8+ ratio) |
|  | Immunoglobulin and mucosal immune markers, including secretory immunoglobulin A (SIgA), immunoglobulin A (IgA), immunoglobulin G (IgG), and immunoglobulin M (IgM) |
|  | Inflammatory cytokine levels, including interleukin-1 (IL-1), interleukin-1 beta (IL-1β), interleukin-2 (IL-2), interleukin-6 (IL-6), interleukin-8 (IL-8), and tumor necrosis factor-alpha (TNF-α) |
|  | Mucosal repair and remodeling biomarkers, including matrix metalloproteinase-9 (MMP-9), vascular cell adhesion molecule-1 (VCAM-1), and epidermal growth factor (EGF) |

The laboratory and biomarker outcomes are considered exploratory outcomes and may be assessed only when relevant to the study design, intervention mechanism, and available resources. Measurement instruments for these outcomes will be selected in a subsequent stage after consensus is reached on what should be measured.
